# Case Report: COVID-19 with cytokine storm in a 16-year-old patient: if heart failures comes think about levosimendan

**DOI:** 10.12688/f1000research.50782.1

**Published:** 2021-03-26

**Authors:** Veronica Rodriguez-Garcia, Jose Luis Guerrero Orriach, Daniel Ariza Villanueva, Jose Manuel Garcia Pinilla, Ainhoa Robles Mezcua, Manuel Rubio Navarro, Jose Cruz Mañas

**Affiliations:** 1 Department of Anaesthesia, Virgen de la Victoria University Hospital, Malaga, Spain; 2 ANESTHESIA-CARDIOLOGY DEPARTMENT, Malaga, Spain; 3 Department of Pharmacology and Pediatrics, School of Medicine, University of Malaga, Malaga, Spain; 4 Department of Cardiology, Virgen de la Victoria University Hospital, Malaga, Spain

**Keywords:** COVID-19, myocarditis, multiorganic failure, levosimendan, case report.

## Abstract

**Introduction: **Our case is unique because the differential diagnosis was a challenge. At first, the patient presented with septic shock and multi-organ failure in the context of a suspected lymphoproliferative syndrome. Once the lymphoproliferative process had been ruled out, hemophagocytic syndrome due to COVID-19 infection was suspected, so he is probably one of the few patients with such an exhaustive study that could contribute to our understanding of COVID-19. We followed therapeutic guidelines that differ from the usual, using adrenalin and levosimendan. Corticosteroids helped to modulate the cytokine storm.

**Case report: **A 16-year-old adolescent was admitted to the intensive care unit with fever, diarrhea, multiorgan failure and septic shock. He was IgG positive for COVID-19 and IgM negative. Thoraco-abdominal computed tomography demonstrated multiple para-aortic and peri-pancreatic lymphadenopathy and acute respiratory distress syndrome. The first suspected diagnosis was a lymphoproliferative syndrome and bacterial infection. The second possibility was a hemophagocytic syndrome in a patient recovering from COVID-19. He was treated with broad spectrum antibiotics because the differential diagnosis was difficult, and we removed them when the microbiological screening was negative. During the course of the disease he presented with severe biventricular dysfunction, probably due to the cytokine storm, so we used inotropic drugs (adrenaline, levosimendan). Infection with Salmonella species group B was diagnosed later, when the patient was in the Internal Medicine ward, although he was asymptomatic.

**Conclusion**: The severity of COVID-19 infection ranges from mild to severe, causing serious disease in some people. Although the pathophysiology is not well known, it seems that in some cases an immune storm is triggered, and it is related to more serious and prolonged disease. In our case, heart failure was important, because it could have worsened the prognosis. Fortunately, the response to levosimendan and corticosteroids was adequate and he recovered favorably until discharge.

## Introduction

The first cases of acute respiratory syndrome caused by COVID-19 were diagnosed in Hubei, China, in December 2019. The high rate of infectivity of the microorganism has triggered a pandemic. Symptoms include dry cough, headache, dyspnea, diarrhea, or fever. However, COVID-19 may also cause respiratory failure, kidney failure, cardiac injury, and central nervous system damage. Patients with comorbidities such as hypertension, obesity, or diabetes are at a higher risk of developing severe symptoms ^1^.

Although the pathophysiology of the virus remains unknown, several studies have associated COVID-19 with a cytokine storm quite similar to that occurring in hemophagocytic syndrome (macrophage activation syndrome). This syndrome is characterized by elevated levels of interleukins (IL-1b, IL-6, IL-10, IL-12), interferon (alpha, gamma), and tumor necrosis factor (TNF-alpha); hypertriglyceridemia; hyperferritinemia; hemophagocytosis in the bone marrow, cerebrospinal fluid, or lymph nodes; and manifests in the form of fever, hepatosplenomegaly, hemorrhagic diathesis, cutaneous rash and alterations of consciousness ^2^.

The myocardial depressant effect of cytokines, an oxygen deficit generated by a prothrombotic state and coronary vasospasms could cause cardiac injury and dysfunction in other organs.

IL-6, cardiac troponins (cTnI/T), and the amino-terminal fraction of the cerebral natriuretic propeptide (NT-proBNP) have been documented to be elevated in patients with cardiac failure secondary to COVID-19 ^3^.

Viral myocarditis is a widely described condition, with symptoms such as heart failure. It usually develops within one to three weeks of COVID-19 infection. Potential COVID-19 myocarditis therapies are based on inotropic drugs and extracorporeal life support ^4^. Levosimendan should be valued as a useful therapy in this type of patients due to its inotropic effects maintained over time and its associated organ protective effects ^5^, which are essential for cardiocirculatory support in cases such as the one reported in this paper.

Our case is unique because the differential diagnosis was quite difficult and interesting. First, it seemed the patient had septic shock and a lymphoproliferative syndrome. Then we thought about hemophagocytic syndrome as well, so he is probably one of the few patients with such an exhaustive study at the immune and hematological level, which could help to understand COVID-19.

Besides that, we follow therapeutic guidelines that differ from the usual, using levosimendan as an inotropic agent ^6^ with very good results and corticosteroids ^7^ without tocilizumab ^8^ due to suspected hemophagocytic syndrome, which helped to modulate the autoimmune response due to the cytokine storm without the need for more immunosuppression with monoclonal antibodies.

## Case presentation

### Patient information

The patient was a 16-year-old Spanish adolescent, Caucasian, with no allergies, with extrinsic asthma treated with corticosteroids and aerosols occasionally. He has no relevant past interventions and no medical, family or psychosocial history (including genetic) of interest. He presented with general discomfort, asthenia and fever (38°C) for three days and was treated with paracetamol 1g/8 hours and azithromycin 500mg/24 hours for three days at home. After seven days, he came to the emergency department with persistence of symptoms, shortness of breath, arthromyalgia and diarrhea.

### Clinical findings

Physical examination revealed a bad general condition. Blood pressure was 70/30 mmHg, heart rate was 110 bpm, respiratory rate was 18 bpm, and axillary temperature was 38.5°C. Lung auscultation found bibasilar crackles and heart auscultation found no murmurs. He had hepatosplenomegaly, oliguria, no edemas, no cutaneous rash and no arthritis. He had diuresis of 170 ml in 12 hours. The results of laboratory testing were: white blood cell count 10200 ^9^/L, with 84.2% neutrophils, red blood cell count 4.2 ^12^/L, hemoglobin 12.5 g/L, platelet count 104000 ^9^/L, serum C-reactive protein level 269.4 mg/L, alanine aminotransferase level 81 U/L, aspartate aminotransferase level 71 U/L, total bilirubin level 3.30 mg/dL, direct bilirubin level 2.73 mg/dL, cardiac troponin I 1135 pg/ml, blood urea nitrogen 48 mmol/L, creatinine 2.72 μmol/L, erythrocyte sedimentation rate of 12 mm/h, procalcitonin 1.96 ng/ml., arterial pH of 7.32, pCO2 of 50.2 mmHg, pO2 of 68 mmHg, HCO _3_ 23 mmol/L, SO2 94%, lactic acid 2.2 mmol/L, ferritin 655.4 ng/mL, triglycerides 161 mg/dL, uric acid 11.3 mg/dL, prothrombin activity 64.7%, international normalized ratio 1.28, activated partial thromboplastin time (aPTT) 28.9 seconds, aPTT ratio 1.16 and D-dimer 7.500 ng/ml. The evolution of laboratory parameters during admission to the Intensive Care Unit (ICU) is shown in Table 1.

**Table 1.** Sepsis parameters.

	ICU admission	Three days after admission	Six days after admission	ICU discharge
Leukocytes	10600 μL ^−1^	31280 μL ^−1^	25320 μL ^−1^	12600 μL ^−1^
Neutrophils	8720 μL ^−1^	27730 μL ^−1^	21190 μL ^−1^	9348 μL ^−1^
CRP	252.3 mg.l ^-1^	92.9 mg.l ^-1^	46 mg.l ^-1^	12.2 mg.l ^-1^
Procalcitonine	1.96 ng.l ^-1^	23.22 ng.l ^-1^	0.27 ng.l ^-1^	0.1 ng.l ^-1^
Lactic acid	2.2 mmol.l ^-1^	2.7 mmol.l ^-1^	1.1 mmol.l ^-1^	1.2 mmol.l ^-1^
Hemoglobin	13.2 g.dl ^-1^	9.4 g.dl ^-1^	10.5 g.dl ^-1^	10.2 g.dl ^-1^
Platelets	105000 μL ^−1^	248000 μL ^−1^	584000 μL ^−1^	874000 μL ^−1^
Ferritin	655.4 ng.ml ^-1^	402 ng.ml ^-1^	497 ng.ml ^-1^	521.6 ng.ml ^-1^
D-dimer	7172 ng.ml ^-1^	4103 ng.ml ^-1^	2724 ng.ml ^-1^	833 ng.ml ^-1^
Fibrinogen	546.8 mg.dl ^-1^	316.8 mg.dl ^-1^	323.7 mg.dl ^-1^	286.7 mg.dl ^-1^
LDH	188 U.l ^-1^	359 U.l ^-1^	248 U.l ^-1^	136 U.l ^-1^
CK	22 U.l ^-1^	49 U.l ^-1^	34 U.l ^-1^	15 U.l ^-1^
Troponin I	1135 ng.l ^-1^	308.4 ng.l ^-1^	130.3 ng.l ^-1^	12.3 ng.l ^-1^
Creatinine	2.72 mg.dl ^-1^	1.41 mg.dl ^-1^	0.65 mg.dl ^-1^	0.61 mg.dl ^-1^
Bilirubin	2.9 mg.dl ^-1^	0.8 mg.dl ^-1^	0.7 mg.dl ^-1^	0.5 mg.dl ^-1^

ICU, intensive care unit; CRP, C-reactive protein; LDH, lactate dehydrogenase; CK, creatine kinase.

### Diagnostic assessment

A nasopharyngeal swab and reverse transcriptase-polymerase chain reaction (RT-PCR) for COVID-19 was negative, but COVID-19 serology was IgM negative and IgG positive. Microbiological screening (urine, blood, sputum and stool culture), serological tests (virus, parasites, fungi and bacteria), and a Mantoux test were carried out. His thoracoabdominal CT scan showed multiple para-aortic and peri-pancreatic lymphadenopathy (compatible with lymphoproliferative syndrome as the most likely diagnosis) and acute respiratory distress syndrome (ARDS). There was no sign of pulmonary thromboembolism, but there were signs of pulmonary hypertension. He was admitted to the ICU and treated with broad spectrum antibiotics (imipenem 1g/8h, linezolid 600mg/12h) and a low dose of norepinephrine (0.05 mcg/kg/min).

An investigation of lymphoproliferative syndrome considering the possibility of hemophagocytic syndrome secondary to COVID-19 was carried out, including autoimmune tests. Protein electrophoresis showed an inflammatory pattern and a direct Coombs test was negative. A bone marrow biopsy showed reactive cells, without atypical cellularity, discarding lymphoproliferative or hemophagocytic syndrome. Tests for autoimmune diseases revealed a low level of C3 and positivity for lupus anticoagulant. The rest of the antibody tests were negative.

Microbiological screening, serological test and Mantoux were all negative, supporting the theory of an exaggerated systemic inflammatory response due to COVID-19. Septic shock was ruled out, so we decided to withdraw antibiotics. Only a rectal swab was positive for COVID-19.

During his time in hospital, the patient presented with acute confusion and agitation. He had no meningeal signs on examination. Cranial CT scan was normal and symptoms remitted within 24 hours.

An electrocardiograph showed atrial fibrillation and diffuse T wave inversion. Transthoracic echocardiography (TTE) was performed regularly, due to elevated cardiac enzymes. The first TTE was normal, but on the third day of admission demonstrated moderate biventricular dysfunction that progressed to severe in the following 24 hours, and atrial fibrillation. The evolution of echocardiographic parameters during admission to the ICU is shown in Table 1.

### Therapeutic intervention

From the beginning our patient was treated with a nasal cannula at 3 liters/minute for five days and a venturi mask at 40% FiO2 for four days. Also, broad spectrum antibiotics (at the beginning, 1g/8h imipenem and 600mg/12h linezolid for six days, and on the third day of admission, 500mg/24h daptomycin and 200mg/24h fluconazole were added to the regimen) and a low dose of norepinephrine (0.05 mcg/kg/min) were administered.

When the possibility of a septic shock was ruled out, antibiotics were withdrawn and 8mg/24 hours dexamethasone was started to try to contain the cytokine storm due to COVID-19. The dose was increased to 12 mg when he suffered with confusion, agitation and heart failure. The patient was treated with adrenalin up to 0.1 mcg/kg/min, 12.5mg/24 hours levosimendan and 20mg/8-12 hours furosemide for eight days. A bolus of 150 mg amiodaron followed by an infusion of 600mg in 24 hours was administered when atrial fibrillation was targeted. He was given enoxaparin 60mg/24 hours during the first days and then it was increased to 60mg/12 hours.

A new TTE that was performed four hours after levosimendan was initiated showed a moderate improvement in left ventricular function and a slight improvement in right ventricular function. In 12 hours, the cardiac function returned to normal and sinus rhythm was recovered ( Table 2).

**Table 2.** Echocardiographic parameters.

	Day 3	Day 4	4 hours after levosimendan	12 hours after levosimendan	24 hours after levosimendan	ICU discharge
LVEF	46%	32%	40%	45%	50%	65%
LVIDD	59 mm	56 mm	Normal	Normal	53 mm	53 mm
SPAP	55-60 mmHg	45 mmHg	40 mmHg	40 mmHg	35 mmHg	35 mmHg
S Wave	15 cm/seg	8 cm/seg	12 cm/seg	15 cm/seg	15 cm/seg	15 cm/seg
TAPSE	25 mm	11 mm	17 mm	23 mm	23 mm	17 mm

LVEF, left ventricular ejection fraction; LVIDD, left ventricular internal diameter in diastole; SPAP, pulmonary arterial systolic pressure; S wave; systolic wave; TAPSE, tricuspid annular plane systolic excursion; ICU, intensive care unit.

### Outcomes

Hemodynamic support therapy was withdrawn, and oxygen therapy, furosemide and dexamethasone were progressively reduced. He didn´t have any adverse events with the medication; it was well tolerated.

In a few days, the clinical picture resolved and the patient was discharged to the Internal Medicine ward. The evolution of his condition was very favorable. He was afebrile, hemodynamically stable, with good food tolerance and without other clinical symptoms. Microbiological screening was repeated by Internal Medicine and a stool culture was positive for group B Salmonella species. The patient had no symptoms, but ciprofloxacin was administered for seven days and then he was discharged hemodynamically stable, eupneic and with oxygen saturation of 99% with oxygen supply of 0.28%. Two weeks later he left the hospital.

A timeline with information from the current episode of care is shown in Table 3.

**Table 3.** Timeline with information from the current episode of care

Initial patient assessment	- A 16-year-old Spanish adolescent, Caucasian, no allergies, extrinsic asthma treated with corticosteroids and aerosols occasionally. - No relevant past interventions and no medical, family or psychosocial history (including genetic) of interest.
	- Blood pressure 70/30 mmHg, heart rate 110 bpm, respiratory rate 18 bpm, axillary temperature 38.5°C. - Lung auscultation: bibasilar crackles. - Heart auscultation without murmurs. - Hepatosplenomegaly, oliguria, no edemas, no cutaneous rash and no arthritis. - Diuresis 170 ml in 12 hours.
	- General discomfort, asthenia and fever (38°C) for three days. - He was treated with paracetamol and azithromycin at home. - Six days after, he came to the emergency department with persistence of symptoms, shortness of breath, arthromyalgia and diarrhea.
Diagnostic evaluation and therapeutic interventions	- Nasopharyngeal swab RT-PCR and serology of COVID-19. - Blood test, protein electrophoresis. - Thoracoabdominal CT scan. - Microbiological screening and serological test. - Bone marrow biopsy. - Autoimmune test. - Cranial CT scan. - Electrocardiograph, transthoracic echocardiography.
	- Paracetamol 1g/8h and azithromycin 500mg/24h for three days. - Broad spectrum antibiotics (imipenem 1g/8h, linezolid 600mg/12h for six days). Daptomycin 500mg/24h and fluconazole 200mg/24h were added on the third day of admission. - Nasal cannula 3 liters/minute for five days and then venturi mask at 40% for four days. - Norepinephrine 0.05 μcg/kg/min for four days. - Dexamethasone 8mg/24h for three days and later 12mg/24h for four days. - Enoxaparin 60mg/24h for three days and then 60mg/12h until discharge. - Adrenalin 0.1 μcg/kg/min for two days. - Levosimendan 12.5mg in 24 hours. - Furosemide 20mg/8-12h for eight days. - Bolus of amiodaron 150mg, followed of an infusion of 600mg in 24h.
Outcomes and follow-up	- After a good recovery in ICU, the patient was discharged to the Internal Medicine ward. - He was afebrile, hemodynamically stable, without antibiotics and decreasing dosage of corticosteroids. - A new stool culture was positive for group B Salmonella species. - He had no symptoms, but ciprofloxacin was administered for seven days and then he was discharged.
	- In a follow-up visit to the Internal Medicine clinic he remained asymptomatic and without sequelae. - Cardiac magnetic resonance imaging was performed, which was normal.

CT, computed tomography; RT-PCR, reverse transcriptase polymerase chain reaction; ICU, Intensive Care Unit.

## Discussion

The Systemic inflammatory response syndrome (SIRS) may be caused by sepsis of bacterial origin, but sometimes it is difficult to substantiate and the differential diagnosis from non-infectious conditions is frequently a challenge. Definitive diagnosis requires isolation of a microorganism, but occasionally this is not possible ^9^. On the other hand, the ability of some viruses to trigger a secondary autoimmune condition is well known. As we said before, on many occasions it is difficult to find out what has been the true trigger of the SIRS. During the COVID-19 pandemic, cases of cardiac involvement have been described ^10- 12^ and in many cases autoimmune diseases secondary to infection have been triggered. Elevated levels of IL-6 have been detected in these patients, but we are unable to determine them in our hospital ^13, 14^. The diagnosis of the disease can be complicated, and many times it is not possible to have absolute certainty, although due to possible complications, in cases of high suspicion, it is still necessary to treat.

In this case, cardiac failure especially contributed to the severity of the patient’s condition. Myocarditis is an inflammatory disease of the heart muscle caused by viruses mainly, although bacteria, toxic drugs and autoimmune diseases can produce it too. The exacerbation of the inflammatory response along with the cytokine storm and its associated cardio-depressor and prothrombotic effects could be the cause of alterations in the coronary circulation (microcirculation and vasospasm), myocardial dysfunction, and increased oxygen consumption ^15^. Cardiac magnetic resonance imaging is useful for diagnosis but only endomyocardial biopsy can establish the etiological diagnosis ^16^. We did not perform an endomyocardial biopsy due to coagulopathy and the pandemic situation.

The patient was treated with adrenaline, diuretics, and levosimendan. He was also receiving anti-inflammatory treatment with dexamethasone, for suspicion of a possible immunological disease secondary to COVID-19 ^17^. heart function recovered in a couple of days. The benefits of levosimendan in infectious myocarditis have been endorsed by different studies. It has been shown to be superior over dobutamine in terms of mortality in patients with heart failure ^18, 19^. It is a novel drug to treat myocardial dysfunction due to sepsis ^20^, myocardial infarction with left ventricular failure ^21^ or cardiac decompensation ^22^. Its cardioprotective effects are because it causes coronary vasodilation, reduces preloading and postloading, and activates mitochondrial-K+ ATP channels. Its inotropic, coronary, antiplatelet, antiapoptotic, and anti-inflammatory effects increases cardiac output and decreases the ventricular filling pressure, pulmonary and systemic vascular resistance ^23^. This was the reason we decided to use levosimendan, after not getting a full response to adrenaline and the progression of the patient was satisfactory.

When he was discharged to the Internal Medicine ward, he continued to show favorable progress and clinical improvement. However, they repeated the stool culture again and it was positive for Salmonella species and treated with ciprofloxacin, although the patient remained asymptomatic. In subsequent check-ups the patient had no sequelae of the disease and cardiac magnetic resonance imaging was performed, which was normal. This makes us suspect that the Salmonella infection was probably acquired in hospital and it was not the cause of myocarditis, although we cannot completely rule it out.

## Data availability

All data underlying the results are available as part of the article and no additional source data are required.

## Consent

Written informed consent for publication of their clinical details was obtained from the patient.
