# Case Report: COVID-19 with cytokine storm in a 16-year-old patient: if heart failures comes think about levosimendan

**DOI:** 10.12688/f1000research.50782.2

**Published:** 2023-02-07

**Authors:** Veronica Rodriguez-Garcia, Jose Luis Guerrero Orriach, Daniel Ariza Villanueva, Jose Manuel Garcia Pinilla, Ainhoa Robles Mezcua, Manuel Rubio Navarro, Jose Cruz Mañas

**Affiliations:** 1 Department of Anaesthesia, Virgen de la Victoria University Hospital, Malaga, Spain; 2 ANESTHESIA-CARDIOLOGY DEPARTMENT, Malaga, Spain; 3 Department of Pharmacology and Pediatrics, School of Medicine, University of Malaga, Malaga, Spain; 4 Department of Cardiology, Virgen de la Victoria University Hospital, Malaga, Spain

**Keywords:** COVID-19, myocarditis, multiorganic failure, levosimendan, case report.

## Abstract

**Introduction: **Our case is unique because the differential diagnosis was a challenge. At first, the patient presented with septic shock and multi-organ failure in the context of a suspected lymphoproliferative syndrome. Once the lymphoproliferative process had been ruled out, hemophagocytic syndrome due to COVID-19 infection was suspected, so he is probably one of the few patients with such an exhaustive study that could contribute to our understanding of COVID-19. We followed therapeutic guidelines that differ from the usual, using adrenalin and levosimendan. Corticosteroids helped to modulate the cytokine storm.

**Case report: **A 16-year-old adolescent was admitted to the intensive care unit with fever, diarrhea, multiorgan failure and septic shock. He was IgG positive for COVID-19 and IgM negative. Thoraco-abdominal computed tomography demonstrated multiple para-aortic and peri-pancreatic lymphadenopathy and acute respiratory distress syndrome. The first suspected diagnosis was a lymphoproliferative syndrome and bacterial infection. The second possibility was a hemophagocytic syndrome in a patient recovering from COVID-19. He was treated with broad spectrum antibiotics because the differential diagnosis was difficult, and we removed them when the microbiological screening was negative. During the course of the disease he presented with severe biventricular dysfunction, probably due to the cytokine storm, so we used inotropic drugs (adrenaline, levosimendan). Infection with Salmonella species group B was diagnosed later, when the patient was in the Internal Medicine ward, although he was asymptomatic.

**Conclusion**: The severity of COVID-19 infection ranges from mild to severe, causing serious disease in some people. Although the pathophysiology is not well known, it seems that in some cases an immune storm is triggered, and it is related to more serious and prolonged disease. In our case, heart failure was important, because it could have worsened the prognosis. Fortunately, the response to levosimendan and corticosteroids was adequate and he recovered favorably until discharge.

## Introduction

The first cases of acute respiratory syndrome caused by COVID-19 were diagnosed in Hubei, China, in December 2019. The high rate of infectivity of the microorganism has triggered a pandemic. Symptoms include dry cough, headache, dyspnea, diarrhea, or fever. However, COVID-19 may also cause respiratory failure, kidney failure, cardiac injury, and central nervous system damage. Patients with comorbidities such as hypertension, obesity, pulmonary disease or diabetes are at a higher risk of developing severe symptoms. ^1^

Although the pathophysiology of the virus remains unknown, several studies have associated COVID-19 with a cytokine storm quite similar to that occurring in hemophagocytic syndrome (macrophage activation syndrome (MAS)). This syndrome is characterized by elevated levels of interleukins (IL-1b, IL-6, IL-10, IL-12), interferon (alpha, gamma), and tumor necrosis factor (TNF-alpha); hypertriglyceridemia; hyperferritinemia; hemophagocytosis in the bone marrow, cerebrospinal fluid, or lymph nodes; and manifests in the form of fever, hepatosplenomegaly, hemorrhagic diathesis, cutaneous rash and alterations of consciousness. ^2^ MAS may present as frequent complication of Kawasaki disease.

Kawasaki disease ^3^ is a vasculitis characterized by high fever for more than 5 days, erythema on lips or oral or conjunctival mucosa, erythema and desquamation of the hands and feet, lymphadenopathy, cardiac lesions (coronary artery aneurysms), elevated acute phase reactants and exclusion of any other microbial cause, same as hemophagocytic syndrome and other autoimmune diseases. ^4^

On May 2020, the Centers for Disease Control and Preventions (CDC), reported cases of multisystem inflammatory syndrome in children (MIS-C) associated with COVID-19 infection. ^5^ Diagnostic criteria include severe disease requiring hospitalization, fever, laboratory markers of inflammation, multiorgan involvement, positive RT-PCR SARS-CoV-2, antibody test positive during hospitalization or contact with a person infected of COVID-19 within 4 weeks before symptom development. MIS-C also has many similarities with Kawasaki disease. ^3^

Myocardial damage is the leading cause of morbidity and mortality. It is due to cytokines effects and hypoxic conditions rather than SARS-CoV-2 invasion in myocardial cells. ^6^

IL-6, cardiac troponins (cTnI/T), and the amino-terminal fraction of the cerebral natriuretic propeptide (NT-proBNP) have been documented to be elevated in patients with cardiac failure secondary to COVID-19. ^7^

Viral myocarditis of MIS-C is a widely described condition, with symptoms such as heart failure. It usually develops within one to three weeks of COVID-19 infection. Potential COVID-19 myocarditis therapies are based on inotropic drugs and extracorporeal life support. ^8^ Levosimendan should be valued as a useful therapy in this type of patients due to its inotropic effects maintained over time and its associated organ protective effects, ^9^ which are essential for cardiocirculatory support in cases such as the one reported in this paper.

The differential diagnosis was quite difficult and interesting. First, a septic shock and a lymphoproliferative syndrome. Then we thought about MAS as well, so he has an exhaustive study at the immune and hematological level, which could help to understand COVID-19.

Besides that, we follow therapeutic guidelines that differ from the usual, using levosimendan ^10^ as an inotropic agen with very good results and corticosteroids ^11^^,^^12^ which helped to modulate the autoimmune response due to the cytokine storm. Tocilizumab, ^13^ anakinra or immunoglobulins were not used. ^14^^,^^15^

## Case presentation

### Patient information

The patient was a 16-year-old Spanish adolescent, Caucasian, with no allergies, with mild intermittent extrinsic asthma treated with corticosteroids and aerosols (salmeterol 25 mcg/fluticasone 250 mcg two inhalations twice a day). He has no relevant past interventions and no medical, family or psychosocial history (including genetic) of interest. He presented with general discomfort, asthenia and fever (38°C) for three days and was treated with paracetamol 1g/8 hours and azithromycin 500 mg/24 hours for three days at home. After seven days, he came to the emergency department with persistence of symptoms, shortness of breath, arthromyalgia and diarrhea (two diarrheal stools without pathological products).

### Clinical findings

Physical examination revealed a bad general condition. He was dehydrated (dry mucous membranes), but aware, oriented and collaborative. Blood pressure was 70/30 mmHg, heart rate was 110 bpm, respiratory rate was 18 bpm, SpO _2_ 98% and axillary temperature was 38.5°C. Lung auscultation found bibasilar crackles and heart auscultation found no murmurs. He had hepatosplenomegaly, oliguria, no edemas, no cutaneous rash and no arthritis. He had diuresis of 170 ml in 12 hours. The results of laboratory testing were: white blood cell count 10200 μL ^−1^ (4000-11500 μL ^−1^), with 8420 (1800-8000 μL ^−1^) neutrophils, hemoglobin 12.5 g.dl ^−1^ (13-17.5 g.dl ^−1^), platelet count 104000 μL ^−1^ (140000-450000 μL ^−1^), serum C-reactive protein level 269.4 mg.l ^−1^ (<5 mg.l ^−1^), alanine aminotransferase level 81 U/L (7-49 U/L), aspartate aminotransferase level 71 U/L (13-40 U/L), total bilirubin level 3.30 mg.dl ^−1^ (0.2-1 mg.dl ^−1^), direct bilirubin level 2.73 mg.dl ^−1^ (<0.3 mg.dl ^−1^), high sensitiviry cardiac troponin I 1135 ng.l ^−1^, blood urea nitrogen 48 mg.dl ^−1^ (20-50 mg.dl ^−1^), creatinine 2.72 mg.dl ^−1^ (0.7-1.3 mg.dl ^−1^), erythrocyte sedimentation rate of 12 mm/h (<15 mm/h), procalcitonin 1.96 ng.ml ^−1^ (<0.1 ng.ml ^−1^), arterial pH 7.32 (7.35-7.45), pCO _2_ 50.2 mmHg (35-45), pO _2_ 92 (75-100) mmHg, HCO _3_ 23 mmol/L (22-26 mmol/L), SpO _2_ 98% (96-100%), lactic acid 2.2 mmol.l ^−1^ (0.5-2 mmol.l ^−1^), ferritin 655.4 ng.ml ^−1^ (22-322 ng.ml ^−1^), triglycerides 161 mg/dL (22-150 mg/dL), uric acid 11.3 mg/dL (4.4-7.6 mg/dL), prothrombin activity 64.7% (70-130%), international normalized ratio 1.28 (0.8-1.2), activated partial thromboplastin time (aPTT) 28.9 seconds (20.6-31 seconds), aPTT ratio 1.16 (0.8-1.2) and D-dimer 7.500 ng/mL (220-500 ng/mL). The evolution of laboratory parameters during admission to the Intensive Care Unit (ICU) is shown in Table 1.

**Table 1.** Sepsis parameters.

	ICU admission	Three days after admission	Six days after admission	ICU discharge
Leukocytes	10600 μL ^−1^	31280 μL ^−1^	25320 μL ^−1^	12600 μL ^−1^
Neutrophils	8720 μL ^−1^	27730 μL ^−1^	21190 μL ^−1^	9348 μL ^−1^
CRP	252.3 mg.l ^−1^	92.9 mg.l ^−1^	46 mg.l ^−1^	12.2 mg.l ^−1^
Procalcitonine	1.96 ng.l ^−1^	23.22 ng.l ^−1^	0.27 ng.l ^−1^	0.1 ng.l ^−1^
Lactic acid	2.2 mmol.l ^−1^	2.7 mmol.l ^−1^	1.1 mmol.l ^−1^	1.2 mmol.l ^−1^
Hemoglobin	13.2 g.dl ^−1^	9.4 g.dl ^−1^	10.5 g.dl ^−1^	10.2 g.dl ^−1^
Platelets	105000 μL ^−1^	248000 μL ^−1^	584000 μL ^−1^	874000 μL ^−1^
Ferritin	655.4 ng.ml ^−1^	402 ng.ml ^−1^	497 ng.ml ^−1^	521.6 ng.ml ^−1^
D-dimer	7172 ng.ml ^−1^	4103 ng.ml ^−1^	2724 ng.ml ^−1^	833 ng.ml ^−1^
Fibrinogen	546.8 mg.dl ^−1^	316.8 mg.dl ^−1^	323.7 mg.dl ^−1^	286.7 mg.dl ^−1^
LDH	188 U.l ^−1^	359 U.l ^−1^	248 U.l ^−1^	136 U.l ^−1^
CK	22 U.l ^−1^	49 U.l ^−1^	34 U.l ^−1^	15 U.l ^−1^
Troponin I	1135 ng.l ^−1^	308.4 ng.l ^−1^	130.3 ng.l ^−1^	12.3 ng.l ^−1^
Creatinine	2.72 mg.dl ^−1^	1.41 mg.dl ^−1^	0.65 mg.dl ^−1^	0.61 mg.dl ^−1^
Bilirubin	2.9 mg.dl ^−1^	0.8 mg.dl ^−1^	0.7 mg.dl ^−1^	0.5 mg.dl ^−1^

ICU, intensive care unit; CRP, C-reactive protein; LDH, lactate dehydrogenase; CK, creatine kinase. Normal values: CK (94-499 U.l ^−1^), LDH (120-246 U.l ^−1^), fibrinogen (180-450 mg.dl ^−1^).

### Diagnostic assessment

A nasopharyngeal swab and reverse transcriptase-polymerase chain reaction (RT-PCR) for COVID-19 (Cepheid-Xpert ^®^ Xpress CoV-2 plus) was negative, but COVID-19 serology was IgM negative and IgG positive. Microbiological screening (urine, blood, sputum and stool culture), serological tests (virus, parasites, fungi and bacteria), and a Mantoux test were carried out. His thoracoabdominal CT scan showed multiple para-aortic and peri-pancreatic lymphadenopathy (compatible with lymphoproliferative syndrome as the most likely diagnosis) and acute respiratory distress syndrome (ARDS). There was no sign of pulmonary thromboembolism, but there were signs of pulmonary hypertension. He was admitted to the ICU and treated with broad spectrum antibiotics (imipenem 1 g/8 h, linezolid 600 mg/12 h) and a low dose of norepinephrine (0.05 mcg/kg/min).

An investigation of lymphoproliferative syndrome considering the possibility of MAS secondary to COVID-19 was carried out, including autoimmune tests. Protein electrophoresis showed an inflammatory pattern and a direct Coombs test was negative. A bone marrow biopsy showed reactive cells, without atypical cellularity, discarding lymphoproliferative or MAS. Tests for autoimmune diseases revealed a low level of C3 and positivity for lupus anticoagulant. The rest of the antibody tests were negative.

Microbiological screening, serological test and Mantoux were all negative, supporting the theory of an exaggerated systemic inflammatory response due to COVID-19. Septic shock was ruled out, so we decided to withdraw antibiotics. Only a rectal swab was positive for COVID-19.

During his time in hospital, the patient presented with acute confusion and agitation. He had no meningeal signs on examination. Cranial CT scan was normal and symptoms remitted within 24 hours.

An electrocardiograph showed atrial fibrillation and diffuse T wave inversion. Transthoracic echocardiography (TTE) was performed regularly, due to elevated cardiac enzymes. The first TTE was normal, but on the third day of admission demonstrated moderate biventricular dysfunction that progressed to severe in the following 24 hours, and atrial fibrillation. The evolution of echocardiographic parameters during admission to the ICU is shown in Table 2.

**Table 2.** Echocardiographic parameters.

	Day 3	Day 4	4 hours after levosimendan	12 hours after levosimendan	24 hours after levosimendan	ICU discharge
LVEF	46%	32%	40%	45%	50%	65%
LVIDD	59 mm	56 mm	Normal	Normal	53 mm	53 mm
SPAP	55-60 mmHg	45 mmHg	40 mmHg	40 mmHg	35 mmHg	35 mmHg
S Wave	15 cm/seg	8 cm/seg	12 cm/seg	15 cm/seg	15 cm/seg	15 cm/seg
TAPSE	25 mm	11 mm	17 mm	23 mm	23 mm	17 mm

LVEF, left ventricular ejection fraction; LVIDD, left ventricular internal diameter in diastole; SPAP, pulmonary arterial systolic pressure; S wave; systolic wave; TAPSE, tricuspid annular plane systolic excursion; ICU, intensive care unit.

### Therapeutic intervention

From the beginning our patient was treated with a nasal cannula at 3 liters/minute for five days and a venturi mask at 40% FiO _2_ for four days. Also, broad spectrum antibiotics (at the beginning, 1 g/8 h imipenem and 600 mg/12 h linezolid for six days, and on the third day of admission, 500 mg/24 h daptomycin and 200 mg/24 h fluconazole were added to the regimen) and a low dose of norepinephrine (0.05 mcg/kg/min) were administered.

When the possibility of a septic shock was ruled out, antibiotics were withdrawn and 8 mg/24 hours dexamethasone was started to try to contain the cytokine storm. The dose was increased to 12 mg when he suffered with confusion, agitation and heart failure. The patient was treated with adrenalin up to 0.1 mcg/kg/min, 12.5 mg/24 hours levosimendan and 20 mg/8-12 hours furosemide for eight days. A bolus of 150 mg amiodaron followed by an infusion of 600 mg in 24 hours was administered when atrial fibrillation was targeted. He was given enoxaparin 60 mg/24 hours during the first days and then it was increased to 60 mg/12 hours.

A new TTE that was performed four hours after levosimendan was initiated showed a moderate improvement in left ventricular function and a slight improvement in right ventricular function. In 12 hours, the cardiac function returned to normal and sinus rhythm was recovered ( Table 2).

### Outcomes

Hemodynamic support therapy was withdrawn, and oxygen therapy, furosemide and dexamethasone were progressively reduced. He didn’t have any adverse events with the medication; it was well tolerated.

In a few days, the clinical picture resolved and the patient was discharged to the Internal Medicine ward. The evolution of his condition was very favorable. He was afebrile, hemodynamically stable, with good food tolerance and without other clinical symptoms. Microbiological screening was repeated by Internal Medicine and a stool culture was positive for group B Salmonella species. The patient had no symptoms, but ciprofloxacin was administered for seven days and then he was discharged hemodynamically stable, eupneic and with oxygen saturation of 99% with oxygen supply of 0.28%. Two weeks later he left the hospital.

A timeline with information from the current episode of care is shown in Table 3.

**Table 3.** Timeline with information from the current episode of care.

Initial patient assessment	- A 16-year-old Spanish adolescent, Caucasian, no allergies, extrinsic asthma treated with corticosteroids and aerosols occasionally. - No relevant past interventions and no medical, family or psychosocial history (including genetic) of interest.
	- Blood pressure 70/30 mmHg, heart rate 110 bpm, respiratory rate 18 bpm, axillary temperature 38.5°C. - Lung auscultation: bibasilar crackles. - Heart auscultation without murmurs. - Hepatosplenomegaly, oliguria, no edemas, no cutaneous rash and no arthritis. - Diuresis 170 ml in 12 hours.
	- General discomfort, asthenia and fever (38°C) for three days. - He was treated with paracetamol and azithromycin at home. - Six days after, he came to the emergency department with persistence of symptoms, shortness of breath, arthromyalgia and diarrhea.
Diagnostic evaluation and therapeutic interventions	- Nasopharyngeal swab RT-PCR and serology of COVID-19. - Blood test, protein electrophoresis. - Thoracoabdominal CT scan. - Microbiological screening and serological test. - Bone marrow biopsy. - Autoimmune test. - Cranial CT scan. - Electrocardiograph, transthoracic echocardiography.
	- Paracetamol 1 g/8 h and azithromycin 500 mg/24 h for three days. - Broad spectrum antibiotics (imipenem 1 g/8 h, linezolid 600 mg/12 h for six days). Daptomycin 500 mg/24 h and fluconazole 200 mg/24 h were added on the third day of admission. - Nasal cannula 3 liters/minute for five days and then venturi mask at 40% for four days. - Norepinephrine 0.05 μcg/kg/min for four days. - Dexamethasone 8 mg/24 h for three days and later 12 mg/24 h for four days. - Enoxaparin 60 mg/24 h for three days and then 60mg/12h until discharge. - Adrenalin 0.1 μcg/kg/min for two days. - Levosimendan 12.5 mg in 24 hours. - Furosemide 20 mg/8-12 h for eight days. - Bolus of amiodaron 150 mg, followed of an infusion of 600 mg in 24 h.
Outcomes and follow-up	- After a good recovery in ICU, the patient was discharged to the Internal Medicine ward. - He was afebrile, hemodynamically stable, without antibiotics and decreasing dosage of corticosteroids. - A new stool culture was positive for group B Salmonella species. - He had no symptoms, but ciprofloxacin was administered for seven days and then he was discharged.
	- In a follow-up visit to the Internal Medicine clinic he remained asymptomatic and without sequelae. - Cardiac magnetic resonance imaging was performed, which was normal.

CT, computed tomography; RT-PCR, reverse transcriptase polymerase chain reaction; ICU, Intensive Care Unit.

## Discussion

Systemic inflammatory response syndrome (SIRS) ^16^ may be caused by sepsis of bacterial origin, but sometimes it is difficult to substantiate and the differential diagnosis from non-infectious/autoimmune conditions is a challenge. Definitive diagnosis requires isolation of a microorganism, but occasionally this is not possible. ^17^

Although the etiology of many autoimmune diseases is uncertain, it is suspected that they are preceded by an active infection, as Kawasaki disease, MAS or MIS-C after COVID-19. ^18^^,^^19^

We ruled out any type of active bacterial infection and he met criteria for MIS-C diagnosis (severe illness that required hospitalization, age less than 21 years, fever, multiorgan failure, elevated acute phase reactants (lack of IL-6 determination), antibody SARS-CoV-2 IgG positive and a rectal swab positive, while a nasopharyngeal swab and reverse transcriptase-polymerase chain reaction (RT-PCR) were negative. Angiotensin-converting enzyme 2 (ACE2) is expressed in the respiratory system less than in the intestinal epithelia, but respiratory symptoms are usually more severe. In fact, it is described that some patients remained stool positive after respiratory samples were negative, which could explain the results. ^20^

Feldstein *et al.* ^21^ showed that one third of the patients tested negative for SARS-CoV-2 by RT-PCR, but had detectable antibodies and in a small subgroup, an interval of 25 days was reported between the infection for COVID-19 and hospitalization for MIS-C. A considerable proportion of patients were infected at least 1-2 weeks before the beginning of MIS-C.

Despite MIS-C seems to be a rare complication of COVID-19, the reason why it develops in some patients and not in others could be related to differences in the nasal expression/gene polymorphisms of ACE-2 and, the gateway for SARS-CoV-2. ^22^

An alteration in the innate immune response, causes the cytokine storm responsable for the most severe cases. ^23^

Elevated levels of IL-6 have been detected in these patients, but we are unable to determine them in our hospital. In any case, the elevated acute phase reactants found and the clinical manifestations support the suspected diagnosis. ^15^

Congenital heart disease, asthma, obesity, diabetes or neurologic conditions seem to be risk factors for severe COVID-19 illness in children and adolescents. ^24^ Our patient has mild extrinsic asthma, well control under aerosol therapy (salmeterol/fluticasone twice per day). Some studies found asthma as a risk factor for hospitalization and respiratory support ^25^ and others ^26^^,^^27^ did not find association. Moreover there are very few data in childhood and more studies are needed.

During the COVID-19 pandemic, cases of cardiac involvement have been described. Arrhythmias have been observed as well. Although Sars-CoV-2 have been found in myocardial cells, it seems that the mechanism of myocardial damage it is secondary to cytokine storm, hypoxic conditions and not as a direct viral infection. Otherwise, the arrihythmia pathogenesis remains unclear, and an abnormal heart rhythm has been found in recovered patients. ^28^ In this case, cardiac failure contributed to the severity of the patient’s condition. Myocarditis is an inflammatory disease of the heart muscle caused by viruses mainly, although bacteria, toxic drugs and autoimmune diseases can produce it too. The exacerbation of the inflammatory response and its associated cardio-depressor and prothrombotic effects could be the cause of alterations in the coronary circulation (microcirculation and vasospasm), myocardial dysfunction, and increased oxygen consumption. ^29^ Cardiac magnetic resonance imaging is useful for diagnosis but only endomyocardial biopsy can establish the etiological diagnosis. ^30^ We did not perform an endomyocardial biopsy due to coagulopathy and the pandemic situation.

There were no official guidelines for the treatment of MIS-C. The individual intra-hospital protocols at that time were based on symptomatic and supportive treatment and on the use of some immunosuppressive drugs. We did not consider antiviral therapy since the patient had already passed the COVID-19 infection.

The patient was treated with adrenaline, diuretics, and levosimendan. He was also receiving dexamethasone that helped to suppress the cytokine storm and enoxaparin to prevent the risk of thrombosis. We did not use inmunoglobulins or monoclonal antibodies as anakinra because the response to corticosteroids was favorable. ^31^

He was raised in a committee for extracorporeal life support if it had been necessary, but heart function recovered in a couple of days.

The benefits of levosimendan in infectious myocarditis have been endorsed by different studies. It has been shown to be superior over dobutamine in terms of mortality in patients with heart failure. ^32^^,^^33^ It is a novel drug to treat myocardial dysfunction due to sepsis, ^34^ myocardial infarction with left ventricular failure ^35^ or cardiac decompensation. ^36^ Its cardioprotective effects are because it causes coronary vasodilation, reduces preloading and postloading, and activates mitochondrial-K+ ATP channels. Its inotropic, coronary, antiplatelet, antiapoptotic, and anti-inflammatory effects increases cardiac output and decreases the ventricular filling pressure, pulmonary and systemic vascular resistance. ^37^ This was the reason we decided to use levosimendan, after not getting a full response to adrenaline and the progression of the patient was satisfactory.

When he was discharged to the Internal Medicine ward, he was improving. However, they repeated the stool culture again and it was positive for Salmonella species and treated with ciprofloxacin, although the patient remained asymptomatic. In subsequent check-ups the patient had no sequelae of the disease and cardiac magnetic resonance imaging was performed, which was normal. This makes us suspect that the Salmonella infection was probably acquired in hospital and it was not the cause of myocarditis, although we cannot completely rule it out.

## Data availability

All data underlying the results are available as part of the article and no additional source data are required.

## Consent

Written informed consent for publication of their clinical details was obtained from the patient.
