# Supplementary material for: Case Report: COVID-19 with cytokine storm in a 16-year-old patient: if heart failures comes think about levosimendan
Source: F1000Res. 2023 Feb 7;10:244. Originally published 2021 Mar 26. [Version 2] doi: 10.12688/f1000research.50782.2 (PMC10966338; doi:10.12688/f1000research.50782.2)
Supplement: Peer review history

# Reviewer report: Reviewer response for version 2

**DOI:** 10.5256/f1000research.142079.r255464

**Published:** 2024-03-27

**Authors:** Srikanth Umakanthan

**Affiliations:** 1 Pathology unit, Department of Paraclinical Sciences, The University of the West Indies, St. Augustine, Trinidad and Tobago

1. Introduction: Do not start the introduction by stating " our case is unique," which should be added, if necessary, in the conclusion.

 2. Indicate the parameters of diagnosis done for concluding multi-organ failure.

 3. The patient presenting complaints need to be elaborated, keeping in mind the possible differential diagnosis.

 4. The first diagnosis of lymphoproliferative syndrome with those presenting complaints sounds skeptical in this case.

 5. The report lacks images and a proper flow chart.

Are enough details provided of any physical examination and diagnostic tests, treatment given and outcomes?

Partly

Is the case presented with sufficient detail to be useful for other practitioners?

Partly

Is sufficient discussion included of the importance of the findings and their relevance to future understanding of disease processes, diagnosis or treatment?

Partly

Is the background of the case’s history and progression described in sufficient detail?

Partly

Reviewer Expertise:

Histopathology, Molecular genetics, COVID19

I confirm that I have read this submission and believe that I have an appropriate level of expertise to confirm that it is of an acceptable scientific standard, however I have significant reservations, as outlined above.

# Reviewer report: Reviewer response for version 2

**DOI:** 10.5256/f1000research.142079.r255461

**Published:** 2024-03-26

**Authors:** Zhongheng Zhang

**Affiliations:** 1 Department of Emergency Medicine, Sir Run Run Shaw Hospital, Zhejiang University School of Medicine, Hangzhou, China

This is generally an interesting case but I have several comments:

 1. Since COVID-19 is a pandemic there are many such cases that COVID-19 with cytokine storm accompanied by heart failure. Some of them have myocarditis. The case does not add causal relationship between drug intervention and outcome. I would suggest discussion on this point in the context of causal inference ( https://doi.org/10.1016/j.lers.2022.10.002) since the causal association can have significant impact on clinical management.

 2. "Fortunately, the response to levosimendan and corticosteroids was adequate and he recovered favorably until discharge."--we actually do not have evidence to prove these drugs are responsible for the recovery, COVID-19 is self-limited even if no treatment is provided. 

 3. The temporal changes of cytokines and other important myocardial markers can be illustrated with plot. 

 4. "A new TTE that was performed four hours after levosimendan was initiated showed a moderate improvement in left ventricular function and a slight improvement in right ventricular function. "-----such minor improvement can be caused by normal variation and variations between operators/interpretators of the cardiography.

Are enough details provided of any physical examination and diagnostic tests, treatment given and outcomes?

Yes

Is the case presented with sufficient detail to be useful for other practitioners?

Partly

Is sufficient discussion included of the importance of the findings and their relevance to future understanding of disease processes, diagnosis or treatment?

Yes

Is the background of the case’s history and progression described in sufficient detail?

Yes

Reviewer Expertise:

intensive care medicine; critical care

I confirm that I have read this submission and believe that I have an appropriate level of expertise to confirm that it is of an acceptable scientific standard, however I have significant reservations, as outlined above.

# Reviewer report: Reviewer response for version 2

**DOI:** 10.5256/f1000research.142079.r162660

**Published:** 2023-02-21

**Authors:** Hrvoje Jakovac

**Affiliations:** 1 University of Rijeka, Rijeka, Croatia

Authors improved manuscript according reviewers' comments. I don't have any comments and I consider the article suitable for indexing.

Are enough details provided of any physical examination and diagnostic tests, treatment given and outcomes?

Partly

Is the case presented with sufficient detail to be useful for other practitioners?

Yes

Is sufficient discussion included of the importance of the findings and their relevance to future understanding of disease processes, diagnosis or treatment?

Partly

Is the background of the case’s history and progression described in sufficient detail?

Yes

Reviewer Expertise:

COVID-19, heat shock proteins, oncogenesis, autoimmunity, neuroscience

I confirm that I have read this submission and believe that I have an appropriate level of expertise to confirm that it is of an acceptable scientific standard.

# Reviewer report: Reviewer response for version 1

**DOI:** 10.5256/f1000research.53866.r154671

**Published:** 2022-11-03

**Authors:** Hrvoje Jakovac

**Affiliations:** 1 University of Rijeka, Rijeka, Croatia

The authors bring an interesting case of a 16-year-old boy with severe COVID-19. Although the authors claim the cytokine storm was an underlying mechanism, there is no clear evidence that this was the scenario in described patients (the authors did not measure plasma cytokine concentration).

 The following sentence in the Introduction section is not clear: " *The myocardial depressant effect of cytokines, an oxygen deficit generated by a prothrombotic state and coronary vasospasms could cause cardiac injury and dysfunction in other organs*". Can SARS-CoV-2 directly infect the cardiac conduction system and cause arrhythmias? (See and refer to: https://doi.org/10.3390/tropicalmed7030043 ^1^)

 The patient's asthma had been treated with corticosteroids and aerosols occasionally. Please provide the treatment regimen and doses in more detail. Could asthma (and corticosteroids) have been a risk factor for the patient described? Please discuss.

- Was SpO2 measured by pulse oximetry immediately at admission?
- Please provide data on the patient's state of consciousness at admission.
- Were there any physical signs of dehydration in the patient upon admission (skin turgor, dry mucous membranes)?
- Please describe the patient's diarrhea (duration, stool frequency, number of stools).
- Please show normal laboratory values in parentheses for each parameter.
- Please provide data on the kit/device used for RT-PCR.

The lack of nasopharyngeal RT-PCR positivity with rectal swabs being positive is the most intriguing detail, but the authors did not discuss it. Please include it in the discussion. What are the potential causes of such findings? Is there any role of ACE2 distribution (see and refer to: https://doi.org/10.1152/ajplung.00119.2020 ^2^)

 The discussion section should be more comprehensive, including consideration of the mechanisms of cardiac affection and arrhythmias described (see above).

Are enough details provided of any physical examination and diagnostic tests, treatment given and outcomes?

Partly

Is the case presented with sufficient detail to be useful for other practitioners?

Yes

Is sufficient discussion included of the importance of the findings and their relevance to future understanding of disease processes, diagnosis or treatment?

Partly

Is the background of the case’s history and progression described in sufficient detail?

Yes

Reviewer Expertise:

COVID-19, heat shock proteins, oncogenesis, autoimmunity, neuroscience

I confirm that I have read this submission and believe that I have an appropriate level of expertise to confirm that it is of an acceptable scientific standard, however I have significant reservations, as outlined above.

## References

[1] : Detection of SARS-CoV-2 Antigens in the AV-Node of a Cardiac Conduction System—A Case Report. *Tropical Medicine and Infectious Disease* .2022;7(3) : 10.3390/tropicalmed7030043 10.3390/tropicalmed7030043 35324590 PMC8955572

[2] : COVID-19: is the ACE2 just a foe?. *American Journal of Physiology-Lung Cellular and Molecular Physiology* .2020;318(5) : 10.1152/ajplung.00119.2020 L1025-L1026 10.1152/ajplung.00119.2020 PMC720087032233935

# Reviewer report: Reviewer response for version 1

**DOI:** 10.5256/f1000research.53866.r97530

**Published:** 2021-12-09

**Authors:** Cecilia Bonazzetti

**Affiliations:** 1 Department of Infectious Diseases, ASST Fatebenefratelli-Sacco, Luigi Sacco University Hospital, Milan, Italy

This case report is well described, at times almost redundant. The description of the clinical case is interesting and complete in all information. However, the discussion should be revised because it lacks a very important etiological entity talking about this topic which is the MIS-C and the most important works regarding this topic are not even mentioned: Feldstein *et al*., 2020 ^1^, Jiang *et al*., 2020 ^2^.

Are enough details provided of any physical examination and diagnostic tests, treatment given and outcomes?

Yes

Is the case presented with sufficient detail to be useful for other practitioners?

Yes

Is sufficient discussion included of the importance of the findings and their relevance to future understanding of disease processes, diagnosis or treatment?

No

Is the background of the case’s history and progression described in sufficient detail?

Yes

Reviewer Expertise:

Infectious Disease

I confirm that I have read this submission and believe that I have an appropriate level of expertise to confirm that it is of an acceptable scientific standard, however I have significant reservations, as outlined above.

## References

[1] : Multisystem Inflammatory Syndrome in U.S. Children and Adolescents. *New England Journal of Medicine* .2020;383(4) : 10.1056/NEJMoa2021680 334-346 10.1056/NEJMoa2021680 PMC734676532598831

[2] : COVID-19 and multisystem inflammatory syndrome in children and adolescents. *The Lancet Infectious Diseases* .2020;20(11) : 10.1016/S1473-3099(20)30651-4 e276-e288 10.1016/S1473-3099(20)30651-4 32818434 PMC7431129
